# Improving in-hospital diabetes awareness and transition of care by a digitalized diabetes management

**DOI:** 10.1007/s12020-025-04368-8

**Published:** 2025-07-28

**Authors:** Lukas van Baal, Johanna Reinold, Dagmar Fuhrer

**Affiliations:** 1 Department of Endocrinology, Diabetes and Metabolism, University Hospital Essen, University Duisburg-Essen, Essen, Germany; 2 Department of Nephrology, University Hospital Essen, University of Duisburg-Essen, Essen, Germany

**Keywords:** Diabetes, Digitalization, Discharge summaries, Information transfer, Transition

## Abstract

**Purpose:**

Diabetes as common comorbidity is associated with an impaired outcome for inpatients. Inadequate transition of dysglycemia specific information from hospital to outpatient setting may disrupt continuity of care and contribute to impaired patient outcome. We tested whether a digitalized diabetes management improves awareness of in-hospital health care professionals for diabetes as comorbidity.

**Methods:**

SmartDiabetesCare, a digitalized diabetes management, was carried out prospectively on five non-ICU wards as a quality improvement project (QiP SDC) including systematic screening for dysglycemia at admission, flagging of identified cases, continuous glucose monitoring and a proactive diabetes-team. Chance for correctly documented dysglycemia specific information in discharge letters written during QiP SDC in comparison to usual diabetes care (UDC) was calculated and performance was assessed to evaluate the impact of QiP SDC on quality of diabetes-specific transition.

**Results:**

Discharge letters of 1141 cases were included in the analysis. Comparing QiP SDC to UDC, the odds ratio for complete correct transition of diabetes-specific information was 2.80 (95%CI: 1.48–5.29) in QiP SDC vs UDC. Accordingly, the performance score was significantly higher in QiP SDC (1.79 vs. 1.23, *p* < 0.01). If only patients with newly diagnosed dysglycemia were analyzed, OR for correct documentation increased to 9.22 (95%CI: 4.01–18.80) and performance score remained significantly higher in QiP SDC, but decreased compared to the overall population (0.60 vs. 0.16, *p* < 0.01).

**Conclusions:**

A digitalized diabetes management may raise awareness for diabetes in in-hospital health care professionals and improve quality of diabetes data transition potentially increasing the chance for continuity of care.

## Introduction

Diabetes (D) as one of the most common comorbidities in hospitalized patients is associated with an adverse outcome including higher risk for readmission and health care cost [1, 2]. Insufficient communication between hospital-based and primary care physicians at discharge may interrupt the continuity of care, contribute to delay of reaching optimal treatment goals and medication errors and in consequence leading to an adverse outcome [3, 4]. Therefore, current guidelines recommend in-hospital assessment of glycemic status and planning recommendations regarding long-term care, resulting in a dedicated discharge management with transition of diabetes relevant information from in- to outpatient setting [5–9]. However, a considerable gap exists between guideline recommendations and real-world evidence. A large meta-analysis, composed of 55 observational and 18 controlled studies, revealed missing documentation of comorbidities in 83% and of treatment recommendations in 25% of discharge letters [10]. In-hospital diabetes management strategies such as care by a specialized diabetes-team and standardized discharge formats have been demonstrated to improve quality of transition to primary care physicians [5, 11]. We hypothesized that a digitalized in-hospital diabetes-management program offering a standardized documented brief diabetes summary, may facilitate transfer of diabetes-relevant information into discharge letters.

## Material and methods

### Study design

This single-centre retrospective study was conducted as part of the university hospital’s quality improvement project SmartDiabetesCare (QiP SDC) at the University Hospital Essen on five non-ICU wards (department of dermatology, neurology, trauma surgery and infectiology). In brief, a structured digital electronic patient file with a focus on dysglycemia specific information was implemented at these five stations. Diagnosis of dysglycemia was visualized to the in-hospital physician by algorithm-based flagging of the patients on the electronic ward overview. Besides, application of continuous glucose monitoring and cloud-based dysglycemia management, the diabetes-team provided dysglycemia specific information to the in-hospital physician in a glance summary (SDC sheet), which could be copied and pasted in total or partially into the discharge letter. A” Push notification” was set into the patient’s electronic health record (EHR) whenever the diabetes-team updated treatment recommendations and the QiP SDC sheet could be checked within the EHR.

Documentation of glycemic status and regarding treatment recommendations in discharge summaries of the patients were retrospectively correlated between patients supplied with QiP SDC and a usual diabetes care (UDC). Information in UDC were obtained from discharge letters of patient hospitalized in the departments of dermatology, neurology and trauma surgery from 01/SEP/2019 until 31/AUG/2020 and the department of infectiology from 01/NOV/2020 until 08/MAR/2021. Patients who passed away during the hospitalization were excluded from the analysis.

To quantify quality of dysglycemia specific information in the discharge letter, a performance score was introduced to evaluate presence as well as correctness of transferred dysglycemia specific content (Table 1). The following situations were distinguished: i) Mentioning of any dysglycemia related diagnosis (for example diabetes mellitus) resp. any dysglycemia related treatment (for example metformin) received 1 point each. ii) Correct and complete mentioning of diagnosis (for example type 2 diabetes mellitus, actual HbA1c 6.6%) resp. treatment recommendation (for example metformin 1000 mg 1-0-1) received an additional point each resulting in a minimum of 0 and a maximum of 4 points. A score of 0 points was interpreted as “very poor”, 1 as “poor”, 2 as “moderate”, 3 as “good” and 4 as “very good” quality of documentation.

**Table 1:** Transition performance score

	Diagnosis	Treatment recommendation
Absence	0	0
Presence	1	1
Incorrect	0	1
Correct	1	1
Score	Minimal total score: 0 Maximal total score: 4	
Interpretation	0 = very poor, 1 = poor, 2 = moderate., 3 = good, 4 = very good quality	

Score values were compared between discharge letters of patients during QiP SDC and those of patients receiving UDC.

### Description of patients

#### Patient cohort

All patients admitted to wards participating in QiP SDC for dermatological, neurological, trauma surgery or infectious diseases within the observation period were included into the study. Indication for in-hospital care was made by the respective departments of dermatology, neurology, trauma surgery, infectious diseases and emergency medicine.

### Statistical analysis

Data were analyzed using GraphPad Prism (GraphPad Software Inc., San Diego, CA, USA) and SPSS 27.0 (IBM Corporation, Armonk, NY, USA) software. Results are shown as mean ± standard deviation and range or absolute number and percentage affected. A value of *p* < 0.05 was considered statistically significant. Documentation of diagnosis of D and PreD and treatment recommendations in discharge summaries were compared using multinominal logistic regression analysis to calculate OR and 95%CI using documented diagnosis respectively diabetes- and prediabetes-specific treatment recommendation vs. no documentation in discharge summaries as dependent variables and QiP SDC vs. UDC as independent variables. The analysis was performed un-adjusted and adjusted for sex, history of dysglycemia, non-insulin antidiabetic treatment, insulin use, intensity of insulin therapy, ward, Hba1c, type of dysglycemia (D vs PreD) and length of stay (LoS). Linearity was tested assessed using the Box-Tidwell procedure [12]. Bonferroni-correction was applied to all ten terms in the model [13]. All variables were found to follow a linear relationship. Correlations between predictor variables were low (*r* < 0.70), indicating that multicolinearity was not a confounding factor in the analysis. Analysis was performed 1) in the total cohort and 2) in the subcohort of patients with newly detected dysglycemia.

To analyze group differences in performance, univariate ANOVA was computed followed by Bonferroni-corrected posthoc tests. Analyses were performed without adjustment as well as adjusted for sex, history of dysglycemia, non-insulin antidiabetic treatment, insulin use, intensity of insulin therapy, ward, Hba1c, type of dysglycemia (D vs PreD) and LoS (as analysis of covariance, ANCOVA, with Hba1c and LoS as covariate and sex, history of dysglycemia, non-insulin antidiabetic treatment, insulin use, intensity of insulin therapy, ward and type of dysglycemia (D vs PreD) as between-subject factors). Proportions of performance score categories were compared in QiP SDC and UDC using the “N-1” Chi-squared test as recommended by Campbell [14] and Richardson [15].

## Results

### Demographic characteristics

Discharge letters of 1218 inpatients with dysglycemia at participating wards were available. Seventy-seven patients were excluded due to in-hospital death from the analysis. Accordingly, data of 1141 patients (42.2% female, age: 66.1 ± 10.3 years, LoS 10.8 ± 8.8days) were evaluated. Admission was due to 25.9% (*n* = 295/1141) dermatological, 23.5% (*n* = 268/1141) neurological, 18.1% (*n* = 206/1141) trauma surgery and 32.6% (*n* = 372/1141) infectious indications (COVID-19 in 88.7% (330/372) of infectiology cases as an indication for in-hospital care).

52.2% (*n* = 595/1141) of discharge letters were written during QiP SDC and 47.9% (*n* = 546/1141) during UDC. Sex and age of included patients did not differ between QiP SDC and UDC. A detailed description of the population characteristics is presented in Table 2.

**Table 2:** General characteristics of the study population

Variable	UDC	QiP SDC	*p*	Total
n	546	595		1141
Age (years)	65.7 (±9.54)	66.8 (±12.14)	0.78	66.1 (±10.32)
Sex (f:m:d)	221:325:0	260:335:0	0.27	481:660:0
LOS	10.9 ± 9.3	10.7 ± 8.7	0.37	10.8 ± 8.8
History (%)	349 (63.9)	281 (47.2)	<0.01***	630 (55.2)
HbA1c (%)	6.5 ± 0.7	6.5 ± 0.8	0.26	6.5 ± 0.8
Total Diabetes (%)	258 (47.3)	347 (58.3)	<0.01***	605 (53.0)
Newly diagnosed Diabetes (%)	44 (8.1)	79 (13.3)	0.01*	123 (10.8)
Total Prediabetes (%)	288 (52.8)	248 (41.7)	<0.01***	536 (47.0)
Newly diagnosed Prediabetes (%)	153 (28.0)	235 (39.5)	<0.01***	388 (34.0)
NI-antidiabetics (%)	137 (25.1)	191 (32.1)	<0.01***	328 (28.8)
IT	90 (16.5)	192 (32.2)	<0.01***	282 (24.7)
Basal	19 (3.5)	57 (9.6)	<0.01***	76 (6.7)
PIT	28 (5.1)	51 (8.6)	0.02*	79 (6.9)
BBT	43 (7.9)	84 (14.1)	<0.01**	127 (11.1)
Diagnosis and treatment (%)	157 (28.8)	264 (44.4)	<0.01***	421 (36.9)
Diagnosis or Treatment (%)	215 (39.4)	351 (59.0)	<0.01***	566 (49.6)
Diagnosis (%)	196 (35.9)	334 (56.1)	<0.01***	501 (46.5)
Treatment (%)	176 (32.2)	281 (47.2)	<0.01***	457 (40.1)

*UDC* usual diabetes care, *QiP SDC* quality improvement program smartdiabetes care, *f* female, *m* male, *d* diverse, *NI* non-insulin, *BOT* basal supported oral therapy, *SIT* supplemental insulin therapy, *ICT* intensified conventional insulin therapy, *LOS* length of stay, * stands for *p* < 0.05, *** for *p* < 0.001

### Dysglycemia specific patient characteristics

D and PreD were present in 53.0% (*n* = 605/1141) and 47.0% (*n* = 536/1141) of patients. Every fifth diabetes (123/605, 20.3%) and 64.1% of all PreD (388/536) cases were hitherto unknown with a total of 44.8% (511/1141) new dysglycemia diagnosis.

Among people with known D, 54.2% (=328/605) were treated with non-insulin antidiabetics, 34.1% (*n* = 206/605) with multiple daily insulin injections and 12.6% (*n* = 76/605) with basal insulin only. Proportion of patients with newly diagnosed dysglycemia and of patients receiving antidiabetic treatment was significantly higher in QiP SDC. HbA1c did not differ between QiP SDC and UDC (Table 2).

### Discharge letters

Overall, transition of dysglycemia specific information in discharge letters was insufficient in the majority with no mentioning of any dysglycemia specific information in 50.4% (*n* = 575/1141) while a complete and correct mentioning of dysglycemia specific information was found in 36.9% (*n* = 421/1141) of discharge letters. When comparing transfer of information to outpatient clinic during QiP SDC and UDC, we observed complete and correct documentation of diagnosis and treatment of dysglycemia in 44.4% (*n* = 264/595) of QiP SDC discharge letters and in 28.8% (*n* = 157/546) of UDC discharge letters (*p* < 0.01) (Table 2). Conversely, absence of dysglycemia specific information was observed in 41.0% (*n* = 244/595) of QiP SDC but in 60.6% of UDC (*n* = 331/546) discharge letters (*p* < 0.01).

**Fig. 1:**
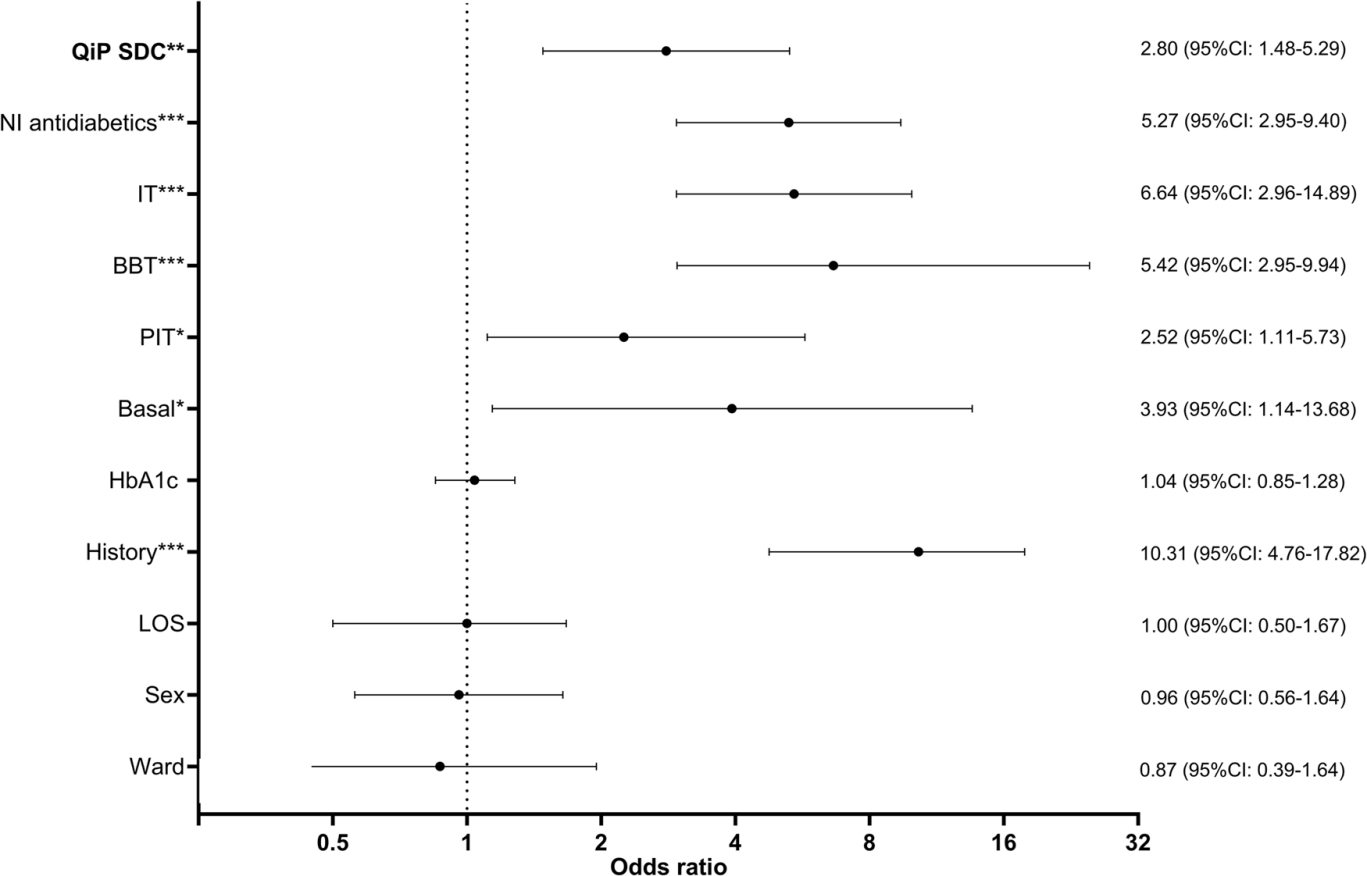
Odds ratio (95%CI) for correct and complete transition of dysglycemia specific information in the study population. LOS Length of stay, PIT prandial insulin therapy, BBT basal bolus therapy, IT insulin therapy, NI Non-insulin, QiP SDC Quality improvement program SmartDiabetesCare, ***: *p* < 0.001, **: *p* < 0.01*: *p* < 0.05

Thus, odds ratio for complete and correct transition of dysglycemia specific information was significantly increased in patients receiving QiP SDC compared to UDC (2.80 (95%-CI: 1.48–5.29); *p* < 0.01, Fig. 1). This applied for both diagnosis as well as treatment specific information (Supplementary fig. 1). Furthermore, an increased OR for transition of dysglycemia specific information could be demonstrated for a known history of diabetes and antidiabetic treatment (Fig. 1, Supplementary fig. 1). When comparing PreD and D groups (data not shown), the proportion of discharge letters with missing information on dysglycemia was slightly albeit not significantly higher in patients with PreD compared to D, with no difference between QiP SDC and UDC.

### Patients with newly detected diabetes and prediabetes

**Fig. 2:**
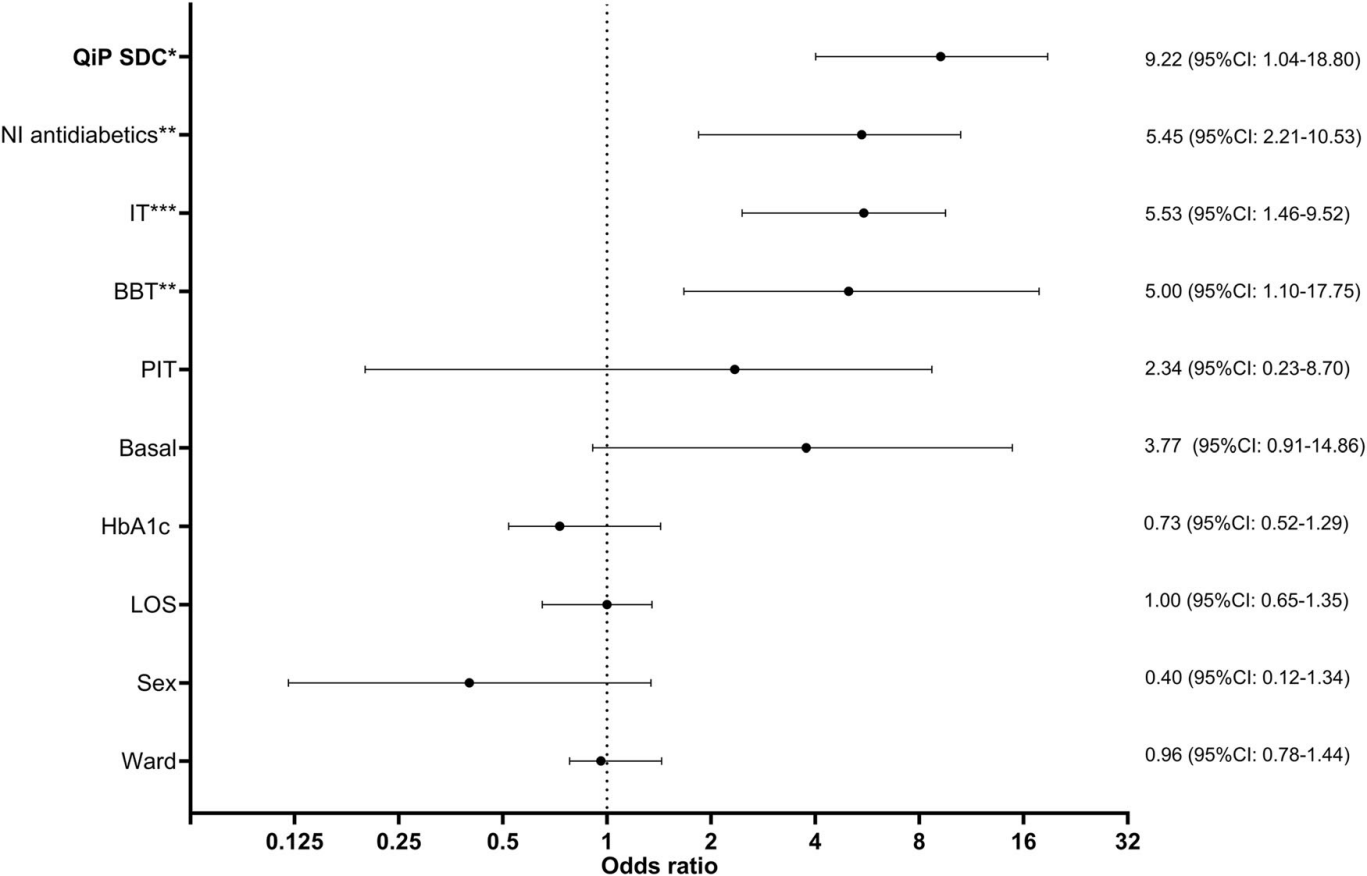
Odds ratio (95%CI) for correct and complete transition of dysglycemia specific information in patients with new-onset Diabetes/Prediabetes. LOS Length of stay, PIT prandial insulin therapy, BBT basal bolus therapy, IT insulin therapy, NI Non-insulin, QiP SDC Quality improvement program SmartDiabetesCare, ***: *p* < 0.001, **: *p* < 0.01*: *p* < 0.05

Odds ratio for complete and correct transition of dysglycemia specific information was significantly increased in patients with newly detected D and PreD receiving QiP SDC compared to UDC ((9.22 (95%-CI: 1.04–18.80); p,04), Fig. 2; Supplementary fig. 2). Besides QiP SDC, an increased odds ratios for transition of dysglycemia specific information were demonstrated for a newly initiated antidiabetic treatment (oral antidiabetic treatment or any kind of insulin therapy) (Fig. 2, Supplementary fig. 2).

### Performance score

Overall mean performance score was 1.52 (±1.62). Proportion of discharge letters with good and very good quality was 35.2% (*n* = 402/1141) compared to 61.2% (*n* = 698/1141) of discharge letters with poor and very poor quality (*p* < 0.01).

**Fig. 3:**
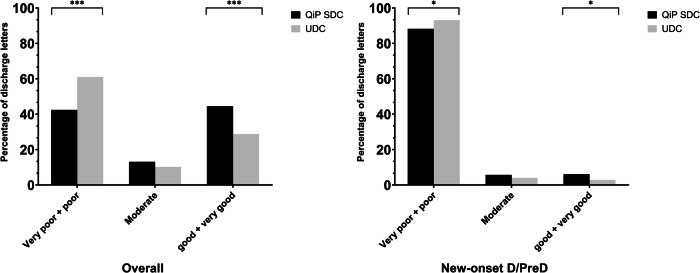
Comparison of performance score regarding dysglycemia specific information in discharge letters of the overall study population and the patients with new-onset dysglycemia. QiP SDC Quality improvement program SmartDiabetesCare, D Diabetes, PreD Prediabetes, *:*p* < 0.05,***: *p* < 0.001

In patients with QiP SDC the overall performance score was significantly higher compared to UDC (1.79 ± 1.60 vs. 1.23 ± 1.60; *p* = <0.01, Table 3). Thus, the proportion of discharge letters of good (=3 points) and very good (=4 points) quality was significantly higher in QiP SDC compared to UDC (44.5% (*n* = 265/595) vs. 28.8% (*n* = 157/546), *p* < 0.01). Conversely, proportion of discharge letters of very poor (=0 points) and poor quality (=1 point) was significantly lower in QiP SDC compared to UDC (42.4% (*n* = 252/595) vs. 61.0% (*n* = 333/546), *p* < 0.01) (Fig. 3).

**Table 3:** Performance score in all patients and only in patients with diagnosis of new-onset dysglycemia

	Overall			New-onset dysglycemia		
	Yes	No	Results of ANOVA	Yes	No	Results of ANOVA
QiP SDC	1.79 ± 1.60	1.23 ± 1.60	*p* < 0.01 F = 45.1	0.60 ± 1.13	0.16 ± 0.58	*p* < 0.01 F = 12.9
History	2.40 ± 1.50	0.43 ± 0.98	*p* < 0.01 F = 133.8	–	–	–
Diabetes	2.66 ± 1.31	0.23 ± 0.74	*p* < 0.01 F = 41.5	1.37 ± 1.39	0.13 ± 0.54	*p* < 0.01 F = 24.6
NI-antidiabetics	3.22 ± 0.94	0.83 ± 1.30	*p* < 0.01 F = 129.0	2.89 ± 0.96	0.34 ± 0.86	*p* = <0.01 F = 70.4
Basal	2.57 ± 0.97	1.44 ± 1.64	*p* < 0.01 F = 32.8	1.65 ± 1.37	0.39 ± 0.94	*p* < 0.01 F = 1.5
IT	2.74 ± 0.69	1.12 ± 1.64	*p* < 0.01 F = 35.8	2.56 ± 0.88	0.29 ± 0.81	*p* = 0.08 F = 3.1
SIT	2.71 ± 0.72	1.43 ± 1.64	*p* < 0.01 F = 34.6	1.67 ± 1.53	0.42 ± 0.98	*p* = 0.03 F = 2.4
BBT	2.74 ± 0.70	1.37 ± 1.64	*p* < 0.01 F = 39.2	2.65 ± 0.79	0.35 ± 0.90	*p* < 0.01 F = 2.7
Sex	1.45 ± 1.63	1.37 ± 1.64	*p* = 0.11 F = 3.6	0.73 ± 1.25	0.28 ± 0.78	*p* = 0.07 F = 6.2

Values are given as mean and standard deviation

*QiP SDC* quality improvement program smartdiabetes care, *NI* non-insulin, *BOT* basal supported oral therapy, *SIT* supplemental insulin therapy, *ICT* intensified conventional insulin therapy, *LOS* length of stay

In patients with newly diagnosed D/PreD mean performance score was significantly higher in QiP SDC compared to UDC (0.60 ± 1.13 vs. 0.16 ± 0.58; *p* = 0.03; Table 3). Moreover, comparison of QiP SDC with UDC revealed, that the proportion of discharge letters of good and very good quality was significantly higher (9.9% (*n* = 31/314) vs. 1.5% (*n* = 3/197) vs.; *p* < 0.01) and proportion of discharge letters of very poor and poor quality significantly lower (76.1%, (*n* = 239/314) vs. 92.9%, (*n* = 183/197); *p* < 0.01) in QiP SDC (Fig. 3). Quality of discharge letters increased from poor to good if an antidiabetic medication was present.

## Discussion

### Summary

This study shows that a proactive digitalized diabetes management significantly enhances the chance of correct documentation of dysglycemia specific information in discharge letters with improved quality of the discharge letters from poor to good. Importantly, the benefit of improved trans-sectoral transition from the in- to the outpatient setting was also evident in patients with newly diagnosed D or PreD.

### Dysglycemia specific discharge management

By analysis of 1141 hospital discharge letters we could demonstrate that the chance of loss of information during transition from in- to outpatient setting is very high with no dysglycemia specific information at all in 50.4% of discharge letters. This pitfall is especially imminent in patients with newly diagnosed D/PreD during hospitalization and truly concerning. Limited literature available indicates similar results [16, 17]. Apart from dysglycemia, however, several studies have described insufficient transfer of relevant information from the in- to the outpatient setting with medication errors occurring in 50–80% and insufficient documentation of diagnoses in 72–82% of discharge letters [5, 18–21]. Importantly, we could demonstrate, that our proactive digitalized diabetes management (QiP SDC) during hospitalization doubled the awareness for correct and complete documentation and transfer of dysglycemia specific information in discharge letters. Furthermore, with our digitalized approach we were able to reduce the documentation errors regarding medication to the lower range of the previously described error rates (52.8%) and regarding diagnoses many times below the previously described error rates (43.9%). This could be due to the fact, that in QiP SDC all dysglycemia specific information were provided in a clearly arranged sheet in the EHR. In this context Doyle et al. demonstrated that a structured discharge summary improves treatment recommendations and documentation of cardiovascular risk factors, as well as diabetes associated complications in discharge letters [22]. Moreover, our proactive digitalized diabetes management included assessment of glycemic status in every patient at admission and early provision of specific care by a specialized diabetes-team. The advantage of such a proactive diabetes-team on non-diabetological wards has been advocated earlier, demonstrating lower rates of documentation errors of dysglycemia specific information during hospitalization and finally in discharge letters [16, 23]. The combination of the aforementioned points in the context of our digital diabetes management ultimately resulted in increased quality of discharge letters from poor to good, both in the overall study population and even more pronounced in the subgroup of patients with new-onset of D and PreD. However, there is still significant need for improvement, even in an academic hospital setting with increased awareness of guidelines and a Smart Hospital Philosophy. These alarming results must be seen in the context of the current burdens of healthcare systems worldwide. Inadequate staffing levels, increasing number of multimorbid patients and rising economic demands on physicians, can adversely effects physicians‘ job performance and in consequence the quality of discharge letters [24, 25]. These problems were even exaggerated through the years of the COVID-19 pandemics [26]. Since our study was conducted during the second COVID-19 wave in Germany, this will likely contribute to the improvable transition of dysglycemia specific information. Nevertheless, we could demonstrate a significant positive impact of our QiP SDC even under these extremely strenuous pandemic condition.

Noteworthy, 79 patients in QiP SDC were newly diagnosed with D. Besides initiation of an antidiabetic treatment in these patients, we could demonstrate that the initiation of antidiabetic treatment, which was only carried out based on our QiP SDC, also increased chance of correct transition of dysglycemia specific information and in consequence quality of discharge letters. The enormous importance of sufficient transition of dysglycemia specific information from the in- to the outpatient care to improve the individual’s outcome in the cohort of patients with newly diagnosed D/PreD is underlined by various studies [27–29].

### Limitations and strengths

While proactive diabetes management was consecutively performed through the same team, discharge letters were written by more than 30 different physicians with differing professions. Therefore, a bias due to interindividual differences cannot be ruled out. However, the population described in our study is to our knowledge the first and biggest cohort with a systematic proactive diabetes-management and analysis of the transition of dysglycemia specific information. Therefore, we offer a precise description of glycaemic status in 1141 patients and can assess the documentation of D/PreD diagnoses and treatment recommendations in discharge letters accurate and reliable. Furthermore, this is the first study to provide insights into both the documentation of medications and diagnoses in discharge letters, and not just one of the two aspects.

## Conclusion

Our study showed, that a digitalized in-hospital diabetes management enhances awareness and supports trans-sectoral transition of care regarding correct and complete documentation of dysglycemia diagnosis and treatment. This benefit could especially be demonstrated in patients with in-hospital newly diagnosed diabetes and prediabetes. More data on further special settings (e.g. perioperative setting, ICU-wards) and long-term follow up investigating possible improvement of glycemic status, are needed to further evaluate the benefit of a digitalized in-hospital diabetes management regarding transition of care.

## Supplementary information

Supplementary Material
