# Supplementary material for: Improving in-hospital diabetes awareness and transition of care by a digitalized diabetes management
Source: Endocrine. 2025 Jul 28;90(2):537–44. doi: 10.1007/s12020-025-04368-8 (PMC12572014; doi:10.1007/s12020-025-04368-8)
Supplement: Supplementary file 1 — Supplementary Material [file 12020_2025_4368_MOESM1_ESM.docx]

Supplementary Figure 1: Overall Odds ratio (95%CI) for correct transition of diabetes specific diagnosis or treatment recommendations, diagnosis, treatment


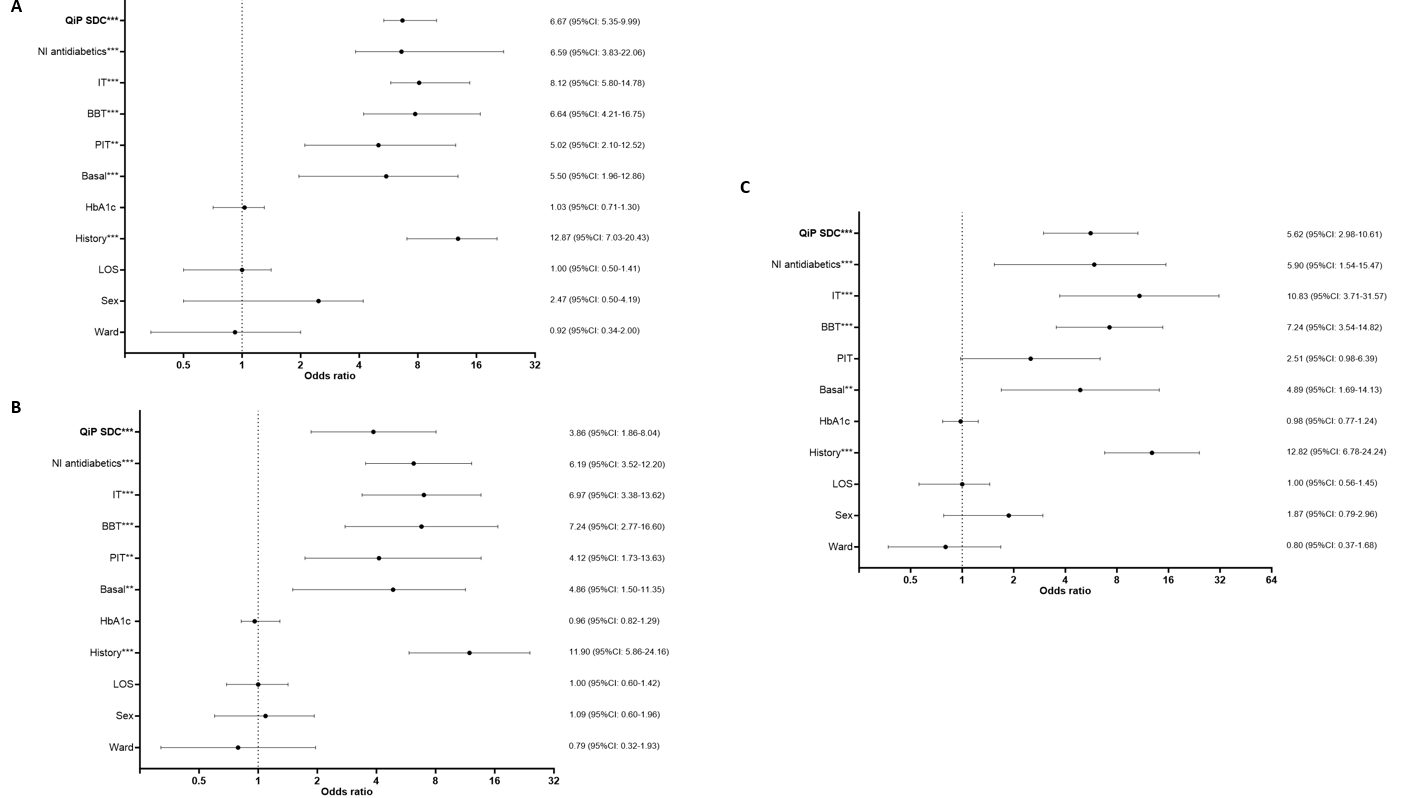


A: Dysglycemia specific diagnosis or treatment, B: Dysglycemia specific diagnosis, C: Dysglycemia specific treatment
LOS: Length of stay, PIT: prandial insulin therapy, BBT: basal bolus therapy, IT: insulin therapy, NI: Non-insulin, QiP SDC: Quality improvement program SmartDiabetesCare, ***: p<.001, **: p<.01*: p<.05

Supplementary Figure 2: Odds ratio (95%CI) for correct transition of diabetes specific diagnosis or treatment recommendations, diagnosis, treatment in patients with new-onset Diabetes/Prediabetes


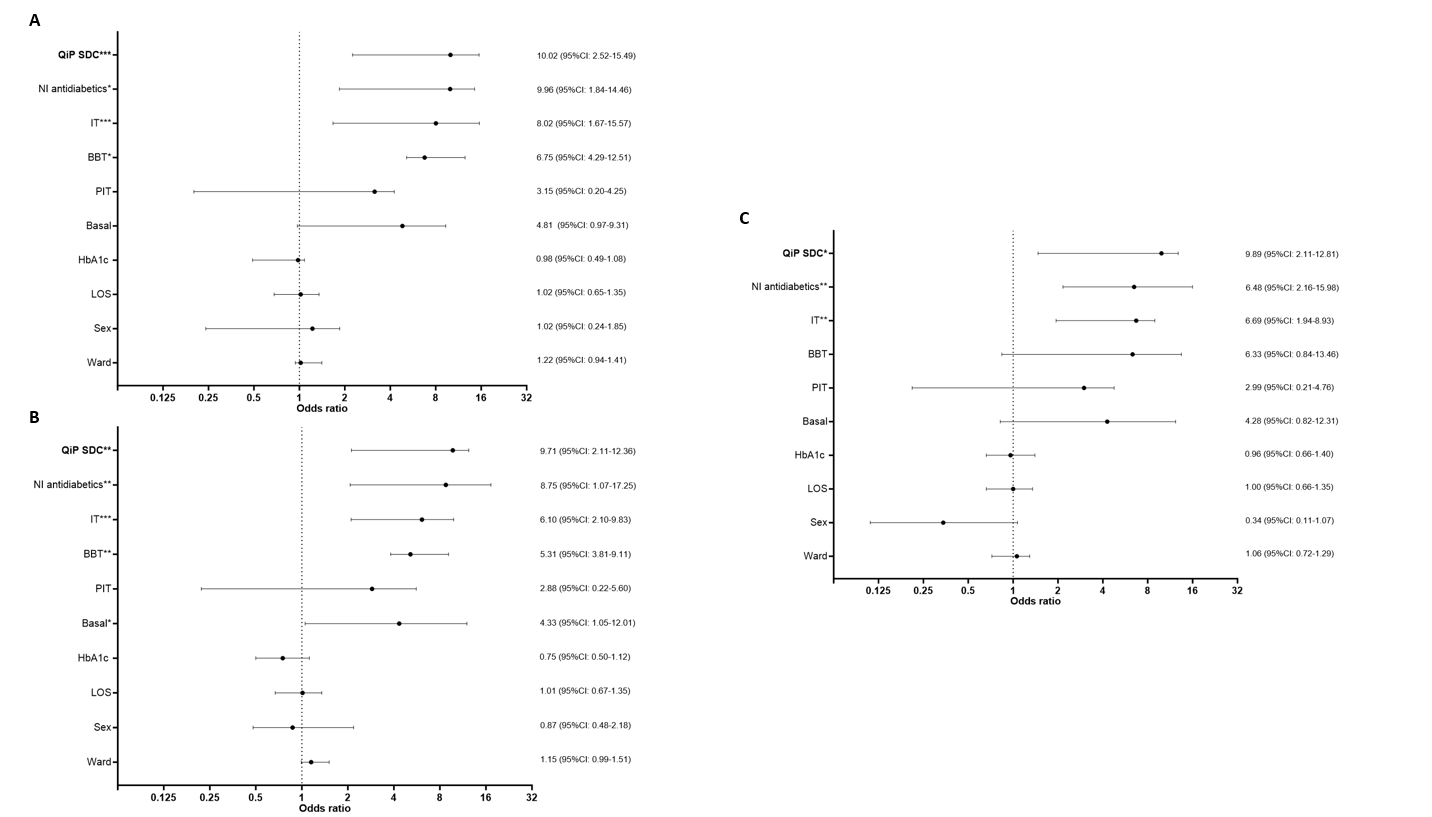


A: Diabetes/Prediabetes specific diagnosis or treatment, B: Diabetes/Prediabetes specific diagnosis, C: Diabetes/Prediabetes specific treatment
LOS: Length of stay, PIT: prandial insulin therapy, BBT: basal bolus therapy, IT: insulin therapy, NI: Non-insulin, QiP SDC: Quality improvement program SmartDiabetesCare, ***: p<.001, **: p<.01*: p<.05
